# Nanotechnology-supported THz medical imaging

**DOI:** 10.12688/f1000research.2-100.v1

**Published:** 2013-03-28

**Authors:** Andreas Stylianou, Michael A Talias

**Affiliations:** 1 Healthcare Management Postgraduate Program, Open University of Cyprus, Nicosia, Latsia, P.O. Box 12794, 2252, Cyprus

## Abstract

Over the last few decades, the achievements and progress in the field of medical imaging have dramatically enhanced the early detection and treatment of many pathological conditions. The development of new imaging modalities, especially non-ionising ones, which will improve prognosis, is of crucial importance. A number of novel imaging modalities have been developed but they are still in the initial stages of development and serious drawbacks obstruct them from offering their benefits to the medical field. In the 21 ^st^ century, it is believed that nanotechnology will highly influence our everyday life and dramatically change the world of medicine, including medical imaging. Here we discuss how nanotechnology, which is still in its infancy, can improve Terahertz (THz) imaging, an emerging imaging modality, and how it may find its way into real clinical applications. THz imaging is characterised by the use of non-ionising radiation and although it has the potential to be used in many biomedical fields, it remains in the field of basic research. An extensive review of the recent available literature shows how the current state of this emerging imaging modality can be transformed by nanotechnology. Innovative scientific concepts that use nanotechnology-based techniques to overcome some of the limitations of the use of THz imaging are discussed. We review a number of drawbacks, such as a low contrast mechanism, poor source performance and bulky THz systems, which characterise present THz medical imaging and suggest how they can be overcome through nanotechnology. Better resolution and higher detection sensitivity can also be achieved using nanotechnology techniques.

## Introduction

Nanotechnology is one of the newest fields of technology and science that has attracted the attention of the scientific community, since it is believed to possess the potential to entirely change our everyday life as we know it up to now. It is quite complicated to identify the origins of nanotechnology; however, no-one can deny the fact that the inspiring lecture of R. Feynman (29 December 1959) “There is plenty of room at the bottom” is the keystone to the field of nanotechnology ^1^. This talk was so amazing for its time that many believe that it represented the birth of the new scientific field of nanotechnology.

Not only are the origins of nanotechnology complicated, but also the definition of the term ‘nanotechnology’ is not as straightforward as it sounds since the field is very recent and there are many conflicting opinions. The first part of the words nanotechnology and nanoscience, the word *nano*, comes from the Greek word ‘nannos’, which means a very short man ^2^ and indicates that we are referring to the technology and science that deal with the physical phenomena/technology in the nanoscale. Generally, we call nanotechnology the manipulation and study of the properties of objects that are, in at least one of their dimensions, smaller than 100 nm.

The importance of nanotechnology is the fact that on the nanometre scale, dimensions of materials are essential to characterise their properties ^3^. At such dimensions, materials possess new physical properties or exhibit new physical phenomena. At such small dimensions, the properties of matter are completely different from what we have been taught and new uncommon properties are observed ^4^. The new properties arise from the fact that at these dimensions the surface area per volume is increased and the material properties obey the rules of quantum mechanics and not the classical physics of the macroscopic scale ^5^. Therefore, nanotechnology has not only to do with small dimensions, but also with new novel physical properties ^2^.

The emerging applications of nanotechnology are so powerful that many scientists believe that it has the potential to radically change the world as we know it and some of them are even wondering whether nanotechnology can push forward the next 'nano-industrial revolution’ ^6, 7^. One area that has been very promising is the application of nanotechnology to medicine ^5^, the so-called *nanomedicine*. Through the developing field of nanomedicine, nanotechnology and medicine come together so that existing therapies and medical techniques can be improved ^8^. Due to its significance for humans, nanomedicine has become one of the most crucial branches of nanoscience. It is considered to be the great challenge of medicine of the 21 ^st^ century, mainly in three key areas: diagnosis, treatment and regenerative medicine ^9^.

A scientific and technological area with so many expectations will inevitably also positively affect the field of medical imaging and radiology. The field of medical imaging is very broad and since the discovery of X-rays, many non-invasive imaging modalities have been invented. Each modality presents its unique characteristics and its intrinsic limitations, and there are differences in their ionising or non-ionising nature, sensitivity, resolution, complexity, time of data acquisition, physical principles, performance conditions, provided information and of course the financial costs. Although the field of medical imaging has a quite long history, new innovative imaging modalities emerge in order to reduce the limitations and expand the capabilities of the existing modalities ^10^. Unfortunately a ‘perfect and ideal’ imaging modality has not yet been developed and the existing modalities are characterised by different limitations. According to Boulaiz *et al.* ^8^, an ideal imaging modality should have a non-invasive nature, high sensitivity and the ability to provide information on cell survival, function and localisation.

An area that attracts the interest of researchers is the use of non-invasive and non-ionising radiation for medical-imaging purposes. It has been stated that there is a revolution in non-invasive imaging modalities ^11^ and imaging modalities that do not use ionising radiation minimise patient’s risks, enable imaging repeatability and in many cases are non-invasive and reduce patient’s suffering. According to Wallace *et al.* ^12^ there is a gap between microscopy and medical imaging and consequently current efforts are focusing on developing non-ionising modalities that can fill this gap. One of the most recent and attractive modalities that satisfy these requirements is Terahertz (THz) imaging ^13– 16^.

**Figure 1.**
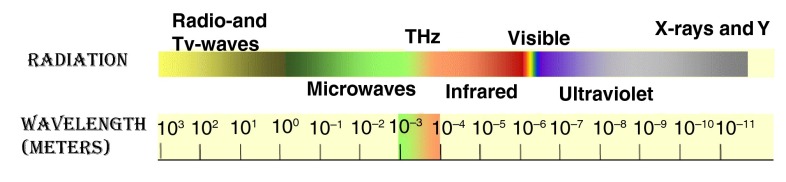
The THz spectrum. It can be seen that THz radiation is the gap between the infrared and microwaves.

THz radiation, also called ‘sub-millimetre radiation’ or ‘T-rays’, is generally defined as the frequency range from 100 GHz to 10 THz ^17^ and is actually the gap between the infrared (IR) and microwaves ^13^ ( Figure 1). This region of the electromagnetic spectrum remained unexplored for many years since there were not appropriate sources (electronic or optical) ^18^ available, although the characteristics of this radiation are unique ( Table 1) and there are a number of potential applications. The development of ultra-short optical pulse lasers and the growth of semiconductor microfabrication techniques pushed for the expansion of THz radiation technology ^19^.

**Table 1.** Characteristics of radiation at 1 THz.

Characteristics	
Period	1 ps
Wavelength	300 μm
Wave number	33 cm ^-1^
Photon energy	4.1 meV
Equivalent temperature	47,6 K

This paper aims to investigate whether nanotechnology can reform a specific imaging modality, THz imaging, and support it in order to overcome its limitations. Generally, it is believed that nanotechnology has the potential to change medicine, and modify it so as to enhance therapy and medical imaging techniques. Many of the proposed applications still remain far beyond what is now possible to achieve using nanotechnology techniques. The purpose of this work is to review the possible applications of not futuristic applications of nanotechnology-supported THz imaging modalities. THz imaging uses non-invasive radiation and although it is among the most attractive emerging imaging modalities, it has not yet become fully established. In order to achieve this, the limitations and drawbacks of the state-of-the-art THz imaging set-ups are first identified. Then nanotechnology-based techniques that were used to improve THz imaging are presented. How THz imaging can help nanotechnology and ethical/risk issues are briefly discussed. The focus is on both the improvement of the current imaging set-ups (detectors, emitting sources, etc.) and the use of nanotechnology-based contrast agents (nano-particles, nano-rods, etc.) to enhance their signals from specific targets.

## Method

In the next section (Background), the current status of THz imaging is briefly presented and the limitations of these unique imaging modalities are presented. In the following section, the results of a systematic review on how nanotechnology can support THz imaging are presented. The searching of relevant material for the review was performed based on electronic resources including the online databases Scopus, Web of Science and PubMed. Google and Google Scholar were used for finding extra material. Furthermore, references found in the initial articles were also used. To capture the relevant studies the keywords and indexing terms that were used included: THz/THz imaging and nanotechnology/nanoscience, nano contrast agents, THz nano-imaging, terahertz nanoscopy. The searching procedure included the following limits: for the nanotechnology-supported THz imaging modalities, only articles published in English between January 2000 and January 2012 were included.

## Background

### General

THz radiation’s applications are expanding so quickly that they have an outstanding potential and social impact ^20^. These applications can be expanded from medical, science and pharmaceutical applications to material non-destructive testing and security purposes. There is special interest in biomedical applications, such as the use of THz radiation as an imaging modality and for spectroscopy studies ^21^, which have the potential for a serious clinical impact ^11^. The first biomedical THz imaging was demonstrated in 1995 ^22^ and since then a new non-invasive imaging modality has emerged.

Since THz radiation has a long wavelength it can penetrate many materials ^23^. Furthermore, polar molecules are sensitive to THz waves and consequently the detection of different hydration levels from tissues can be achieved ^13^. The biomedical applications of THz waves are a consequence of the fact that THz radiation is sensitive to water and, what is more, biological molecules' characteristic energies lie in the THz region ^24^. This is very important considering that water is one of the most important components of the tissue ^25^. Actually, water molecules and all polar liquids absorb all the frequencies in the THz band. As a consequence, THz waves cannot penetrate moist tissue, a fact that enables both the development of imaging set-ups in transition (for *in vitro* studies) and reflection geometry (for *in vitro* and *in vivo* imaging) ^11^. THz radiation is characterised by its ability to penetrate organic materials without ionisation, to distinguish different materials according to their water content and the fact that it can help the clarification of the unknown dynamics in the area of condensed matter physics (e.g. molecular recognition and protein folding) ^26^.

In the case of THz medical imaging, the contrast mechanism arises from the fact that different absorption spectra and refractive indices characterise the different biological tissues when they interact with waves in the THz region ^17^. Consequently, images and information from normal or pathological tissues (with abnormalities) can be obtained. Even from the very first demonstration of imaging from Hu and Nuss ^22^, it was shown that the different water content of two different tissues (porcine muscle and fat) was the contrast mechanism ^12^. Initial studies have demonstrated that biochemical and morphological features of the tissue provide contrast in images formed with THz pulses ^25^.

When THz radiation is used for spectroscopy, the key factor is the fact that the energy of the vibrational and rotation molecules (like proteins and DNA) corresponds to that of the THz photons ^25^. THz spectroscopy can be used to investigate a variety of phenomena of great importance to scientists and engineers ^27^. An interesting biomedical application is the demonstration of using THz spectroscopy to detect mutations and biomolecule conformational changes ^11^. Also, the diagnosis and imaging of cancer is one very promising application of THz imaging technology ^28– 32^. This is a consequence of the fact that there is difference in the water content between healthy tissues and cancerous tumours and, what is more, there are differences due to cell alterations and abnormal protein density alterations that result in larger THz absorption and refractive index ^24, 33^. Although there are many expectations on this technique, the studies seem not to support any clinical application which can achieve high cancer detection rate ^24^.

### THz imaging modalities

Although the field of medical imaging in the last decades has seen great improvements and has significantly contributed towards a better medical practice both for diagnosis and therapy, THz is still developing in all of its components from technological concepts/set-ups to possible applications. THz-material/tissue interactions and the physical/biological mechanisms involved are still not well established and more research is still needed.

The lack of appropriate, low expense and compact-size emitters and detectors of T-rays has meant that the THz band has remained unexplored and unused for many years. Initial inappropriate set-ups were developed with expensive and bulky sources (e.g. free electron lasers, thermal sources), while the detectors demonstrated poor performance, like the liquid helium-cooled bolometers ^11^. In the last decades, a number of novel techniques (like the ultrafast optical switch, the nonlinear method and quantum cascade lasers) innovated the field of THz optoelectronics ^19^. The THz imaging systems can be separated into two main categories: Passive (also named Incoherent) and Active (also named Coherent) Pulsed or Continuous ^34, 35^. Here, we focus on the set-ups of the active categories, since these are the most extensively used for medical imaging purposes, while passive set-ups are most widely used for security purposes in airports and for weapon detection ^36, 37^. Pulsed and continuous THz imaging set-ups are both still in development, but pulsed systems are more widely used ^24^.

At present there are many competitive techniques for generating THz waves (continuous or pulsed). The different THz sources can be separated into three basic categories: electronic sources, photonic sources and quantum cascade lasers. There are also some other smaller categories that are either emerging or not very popular, like p-germanium THz lasers ^38– 40^ and uni-travelling-carrier photodiode ^13, 41^, but they are not the focus here.

There are several ways of detecting THz radiation. There are a large number of published articles explaining the spectrum of the existing detectors and the new principles that are used for detecting T-rays are very broad ^42^. They can be separated into direct detection (with Schottky-barried diodes and bolometers), heterodyne detection (like with super conducting hot electron bolometers) and heterodyne detection that uses photonical-generated THz local oscillators (electronic or photonic mixers) ^43^. Photoconductors (PCs), electro-optic (EO) materials and photodiodes (PDs) are frequently used as photonic mixers. Direct and heterodyne detection are also referred to as incoherent and coherent detection, respectively ^44^. Which method of detection will eventually be applied is mainly determined by the type of THz source that is used in the same set-up ^26^ or which characteristics of the THz waves are intended to be detected.

The THz imaging systems, and THz technology in general, can be separated on whether they uses continuous waves (CWs) or pulses. CW THz is traditionally used for astronomy (like the study of Big Bang radiation), environmental monitoring and plasma diagnostics. Optically Pumped Terahertz Laser is a characteristic CW THz source ^26^. For medical imaging, pulse THz sources are more attractive and have made THz imaging a challenging field for medical imaging.

The term “THz time-domain spectroscopy” (THz-TDS) refers to the technique where THz pulse methods are used for spectroscopy studies ^19^. The same method system can be used for the formation of 2- and 3-D images. THz pulse-imaging is a quite simple methodology and a pump and probe beam are used. The pump beam interacts with the sample, and the detection of the coherent signal is obtained by combining the probe laser with the THz radiation ^25^. The image of the subject can be built up due to the selective absorption of the THz radiation ^26^. Consequently, the detector receives the signals with delay ^26^ and by scanning the sample an image can be formed with each pixel representing the different time-series, which are the different adsorption characteristics ^25^. The obtained data can then be processed with fast Fourier transform so as to move from the time to the frequency domain ^26^.

The characteristics of THz radiation and already developed imaging set-ups also enable the use of this technique as an endoscope-based procedure ^45^. The researchers involved in this study believed that if some limitations could be overcome (like the fact that water and the side-walls of the organs have a similar refractive index and power absorption), THz endoscopes would be a valuable tool for detecting tissue changes within the human body ^45^.

### Limitations of THz imaging

Unfortunately, THz imaging modalities are still characterised by a number of quite significant limitations. Particularly, THz radiation remained unexplored for many years due to the fact that detectors of THz waves were characterised by poor signal-to-noise ratio and slow processing. A second limitation was that the emitters were able to produce only incoherent and low-brightness THz radiation ^25^ and some sources require cryogenic operating temperatures ^20^. The development of both electronic and optical sources that emit at the THz spectrum is difficult to implement but very beneficial ^17^. Some of the initial problems that THz technology has faced have found some solutions but there are also a number of significant drawbacks that still remain ( Table 2) ^23, 46^.

**Table 2.** Advantages and limitations/drawbacks of THz imaging.

Advantages	Drawbacks/limitations
Non-ionising radiation; is considered safe for biological imaging.	Limited penetration depth and THz waves cannot penetrate into the human body due to high water component.
Sensitive to water component. Biological molecules' characteristic energies lie in the THz region (the energy of vibrational and rotational molecules correspond to that of the THz photons).	Difficulties in the development of appropriate THz sources • Measuring speeds and scanning times require improvement. • Bulky systems due to their components, like the use of fs lasers. • System costs are relatively high (mainly due to the use of fs lasers). • Problems in transferring THz waves and difficulties in achieving distance sensing in air over several metres. • Need to improving systems with a large signal-to-noise ratio. • Some THz sources cannot be used at room temperature.
It can perform non-destructive testing and contact-free imaging or characterisation of the sample.	
Compared with microwaves, THz waves possess shorter wavelength and consequently greater spatial resolution can be achieved.	
The long wavelength of the THz photons enables the THz radiation to penetrate many materials.	
THz radiation is not heavily affected by Rayleiyh scattering.	Limited imaging resolution due to long wavelength.
Fills the ‘gap’ in medical imaging modalities.	Applications still in research.
Can perform spectroscopy. Medical imaging can be combined with spectroscopy information. Biochemical and morphological features can be provided simultaneously.	Low contrast between healthy and pathological tissues.

The first drawback of THz imaging is a consequence of the nature of THz waves. Their long wavelength results in limitations in the imaging resolution compared with imaging modalities that use shorter wavelengths ^47^. Due to the long wavelength, THz imaging can illustrate features in the range 1–3 mm, which is not enough for biomedical applications ^11^. The limited penetration depth due to the high body-water component has until now limited possible applications and THz studies to surface tissues such as skin ^48, 49^, teeth ^50^ and the cornea ^51^. The use of the 0.5 THz frequency gives the highest contrast between normal and cancerous tissues, but this minimized the later resolution of THz imaging ^33^. Furthermore, the contrast between healthy and pathological tissues is too low and there is a need for contrast agents ^33^. Humphreys *et al.* ^11^ also stressed the need for well informed and well organised databases that include the different responses of different tissues to THz radiation.

As was shown in the previous section, the use of femtosecond (fs) lasers is a common method for emitting THz radiation in the optical-to-THz conversion. The use of these sources make the commercialisation of THz imaging set-ups difficult due to both the high cost and the bulky dimensions of fs lasers ^17^. This is why quantum cascade lasers are very attractive in the field, since they are expected to be cheaper and of appropriate size. Finally, it must be noted that although the potentials are great, the penetration depth of THz radiation is limited. Consequently, until now research has been concentrated on wound healing, burn diagnosis dermatology and dentistry, where a high depth is not required and the probed tissue is accessible without the need for waveguides ^11, 25^. Furthermore, the majority of the THz applications are still in the research phase, except for a few examples from the TeraView Company (Cambridge, UK), which has developed set-ups and techniques for detecting cancerous cells ^26^. Currently, there are a number of companies that produce THz-related technology including Picometrix Inc. (Michigan, USA), Zomega Terahertz Corporation (New York, USA), Nikon Corporation (Tokyo, Japan), Toptica Photonics (Munich, Germany) and Hamamatsu Photonics (Tokyo, Japan), T-Ray Science Inc. (Vancouver, Canada).

## Nanotechnology for THz imaging

**Figure 2.**
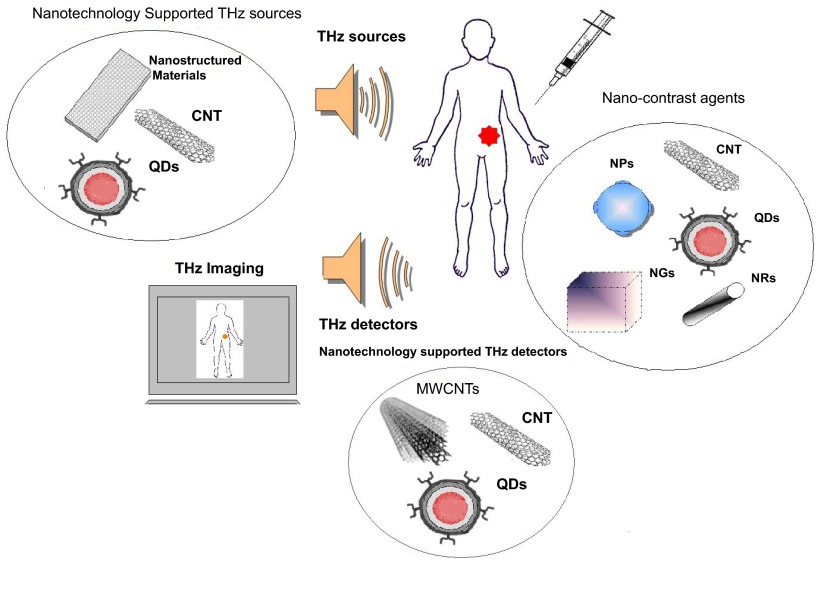
Nanotechnology-supported THz imaging. Nanotechnology methods are used in all the components of THz imaging: contrast agents, sources and detectors (CNT: Carbon Nanotubes, QDs: Quantum Dots, NPs: Nanoparticles, NRs: nanorods, NGs: nanocages, WCNTs: multi-walled CNTs).

As in all the emerging imaging modalities, THz presents some drawbacks that do not allow it to find its place in every day medical use. As was shown, these limitations cover a wide range from the low-performance of emitting sources to the low sensitivity or selectivity to pathological tissues. In order to overcome these drawbacks researchers are expanding their efforts in many different directions. Nanotechnology-based techniques seems to be a crucial key tool in their efforts to improve these imaging modalities. We believe that nanotechnology has the potential to improve the performance of imaging modalities. The next sections aim to discuss how current nanotechnology techniques can directly enhance THz medical imaging modalities. It will be demonstrated that nanotechnology can support THz imaging through several concepts, from using nanoparticles as contrast agents to the development of new THz sources and/or detectors ( Figure 2).

### Nanocontrast agents for THz imaging

One area of THz imaging that nanotechnology could innovate is the use of contrast agents, also called contrast media or probes. Generally, contrast agents are used in order to increase image contrast from healthy and pathological tissue areas or molecules. Many contrast agents have been proposed and used for the existing imaging modalities (e.g. MRI contrast agents) ^52– 54^, but in the case of THz imaging, very few studies have been published. Although this area is in the very early stages, the results are very positive and open new horizons for the clinical application of THz medical imaging. By using contrast agents, it will be possible to enhance the sensitivity of cancer diagnosis by targeting tumours and by enabling the use of higher THz frequencies, which will allow better resolution ^33^.

Nanotechnology can innovate THz imaging in this direction by the use of nanoparticles as contrast agents. With the term 'nanoparticles', a broad range of particles with dimensions in the nanoscale is implied, such as spherical particles, carbon nanotubes (CNT), fullerenes, quantum dots (QDs), cantilevers, nanorods (NRs), nanoshells, nanocages (NGs), nanowires and various metal and metal oxide nanoparticles. Nanoparticles that are characterised by their ability to produce surface plasmons, the so-called plasmon nanoparticles, are particularly interesting as they can be used for imaging and therapy purposes ^55^. Gold (Au) is the most commonly used metal for fabricating nanoparticles for biomedical applications due to its biocompatibility, strong scattering around local surface plasmon (LSP) resonance wavelengths and the ability to accept the bio-conjunction process ^56^.

**Figure 3.**
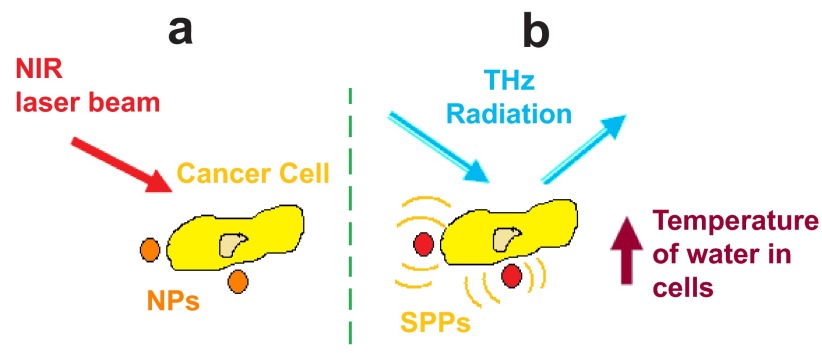
The hyperthermia effect. **a**) First, the cancer cells are probed with nanoparticles (NPs) and are then irradiated with near-infrared (NIR) laser beams. **b**) After irradiation, surface plasma polaritons (SPPs) occur and as a result the temperature of water in the cancer cells is increased. Consequently, the cancer cells can be probed and imaged with THz radiation since the THz signal is sensitive to water temperature alterations.

Nanoparticle contrast agent techniques take advantage of the physical phenomenon known as the hyperthermia effect that occurs due to surface plasma polaritons (SPPs) when near-infrared laser beams irradiate nanoparticles. As a consequence of this phenomenon, the temperature of water in cancer cells (which are probed with nanoparticles) rises and since the THz signal is sensitive to water temperature alterations ^57^, cancer cells can be probed and imaged ( Figure 3).

A significant work on this direction was published by Oh, Son and their colleagues in a series of four recent papers ^33, 58– 60^. The new methodology was called nanoparticle-contrast-agent-enabled terahertz imaging ^58^. Initially, hydroxyapatite gold nanocomposites and gold nanorods (GNRs) were studied and it was shown that contrast agents can enhance sensitivity in THz signals and can be bound in cancer cells and can consequently target cancerous tumours ^33^. Their next *in vitro* experiments were performed in cancer cells with and without GNRs ^58^. Their results showed that although there were not any significant differences in THz reflection images, the enhancement was high under IR irradiation and the differential mode enabled cancer diagnosis. They also demonstrated that tumours could be identified by monitoring the signal at a point without the need of imaging.

Oh *et al.* expanded their experiments *in vivo* by acquiring THz images in tumours of mice 24 hours after the injection of GNRs and the high sensitivity of the differential technique was shown ^59^. Finally, in a recent publication THz molecular imaging (TMI) was demonstrated to be sufficiently sensitive to detect 15 mM of nanoprobes *in vivo* ^60^. What is more, it was characterised in linear proportion to the nanoparticle concentration, which is a very useful quantification property. For their experiments, Oh *et al.* used a reflection-mode THz imaging set-up accompanied by a laser in the IR region for the surface plasma polariton induction ^59^.

Other research groups are also working in the field and Lee *et al.* ^24^ studied gadolinium oxide (Gd _2_O _3_) nanoparticles as possible terahertz imaging contrast agents. Their results demonstrated that these kinds of particles are appropriate for terahertz medical imaging since their interaction with THz waves is very strong (the power absorption is ~3 orders higher than water) ^24^. Moreover, as Gd _2_O _3_ nanoparticles are already used as MRI multi-functional contrast agents, their use for THz studies might enable the combination of the two imaging modalities (MRI-THz) and the combination and enhancement of the offered information.

Apart from the previously mentioned advantages of the use of nanoparticles as nanoprobes (nanocontrast agents), it is believed that they can offer further possibilities. The simultaneous use of the nanoparticles as hyperthermia therapeutic agents and THz imaging can achieve both diagnosis in early stages of cancer ^59^ and therapy. Furthermore, THz imaging techniques can be applied for monitoring drug-delivery processes ^59, 60^ and finally, the use of infrared laser beams for imaging with THz set-ups opens the horizons for real practical THz endoscopy ^58^.

### Nanotechnology-supported new THz sources

One of the most suitable candidates for the development of compact THz sources is the quantum dot (QD) system, although the emission in the THz range has not yet been accomplished. QDs have dimensions between nanometres to a few microns and are characterised by the fact that they contain a tiny droplet of free electrons. Their size, shape and number of electrons can be precisely controlled depending on their possible applications. QDs are very attractive due to their intrinsic discreet energy level. After confirmation of the long carrier relaxation times and the ability to control these times, the way forward for QD-based THz optoelectronic devices was opened ^61^. Takatori *et al.* ^62^ demonstrated that InAc/GaAs (indium monoarsenide/Gallium arsenide) QDs have the potential to be intense terahertz emitters and recently the generation of THz radiation from InAc/GaAs quantum-based photoconductive antennae was achieved ^63^. Moreover, a novel methodology for varying the QD growth parameters for manipulating the band gap in the THz emission range has been demonstrated ^64^. In order to achieve 40 meV differences between intra-bands (E _1_ and E _2_), which is a necessary condition for intra-band THz emission, the effect of growth and monolayer coverage on the energy difference between the ground and excited states of two types of QD structures was investigated ^64^. In a recent publication, a method for generating dual (or multiple) high-power THz difference frequency from a single QD laser diode was demonstrated ^65^.

One other way that nanotechnology can innovate the THz sources, is the novel production of pulsed THz radiation by using nanostructured materials. It has been demonstrated by a group in Glasgow, UK, that appropriate nano-engineered surfaces can enhance THz radiation through surface plasmons (SPs) under a femtosecond laser simulation ^66^. Initially, the emission of terahertz signal due to SPs has been confirmed from metal grating ^66^, nanoparticle (ZnSe) surface ^67^ and nanograin surface (ZnSe) ^68^, while the studies have been expanded to different types of metal surfaces (mainly gold), such as nanoparticles, nanoparticle rings and pyramid-shaped particles ^69^. When light interacts with metallic nanosurfaces, non-linear optical phenomena are enhanced due to the strong interactions and the high field strengths that are produced ^66^. This concept enables the production of a terahertz pulse due to a new process of rectification. Gao and colleagues stated that this phenomenon is a consequence of the electrons' acceleration, which is a result of the surface plasmon excitation and believed that the mechanism is related to the multiphoton photoelectric effect ^69^. In the case of surface nanoparticles, the mechanism of THz emission can be described in terms of dipole orientation ^67^.

Apart from QDs and nanostructure materials, nanotubes, and especially CNTs, is another innovative nanotechnology area that is expected to highly influence THz emission and detection (see next section) of terahertz radiation. CNTs are molecular-scale tubes of graphitic carbon and were invented in 1991 by Iijima at Nec Fundamental Research Laboratories (Tsukuba Science City, Japan) ^70^ and can be used in a variety of ways. Simulation studies have shown that CNT antenna properties can be improved by controlling the length, inter-tube distance and the number of nanotube elements so as to achieve better design of CNT-based sources/detectors for THz studies ^71^. Recently, the traverse vibration of a novel composite NT was studied ^72^. This NT was synthesized by coating CNT with piezoelectric zinc oxide (ZnO), which is a bio-safe and biocompatible material. The results showed than ZnO-CNT can be used for gigahertz/terahertz electromechanical nanoresonators. What is more, the tubular shape of CNT offers sharp tips that are appropriate for field emission, and consequently with the discovery of CNT a new class of field emitters was generated ^73^. CNT bundle arrays have been developed as components of cathodes and have shown very promising results ^73^. The CNTs were arranged as arrays and found to be appropriate for cold cathodes and able to operate at low voltages. It is believed that a high field enhancement, which produces efficient field emission, is caused by rearrangement of the free ends and outliers under an applied field, and is the reason of the bundle arrays' high emission ^73^. Furthermore, in their work Manohara *et al.* ^73^ showed that a highly compact field emission electron gun can be formed with monolithic integration of multiple electrodes. This technique enables electron-beam shaping and a novel miniature electron gun can be fabricated. In an older study, Manohara and colleagues presented the Nanoclystron, which is a novel micro-tube THz source ^74^. In their circuit, the THz emission is achieved by CNTs, which are performing as electron emitters, and silicon-based reflex klystron-type cavities ^74^. The use of highly ordered CNT arrays for THz emission has also recently demonstrated ^73^.

Gamziha *et al.* (2011) are working towards miniaturised vacuum electronic devices that will be able to be used as high power THz sources ^75^. One of the methods they have proposed is nanomaching, which offers many advantages such as rapid prototyping of any circuit. Their recent work on the development of a 0.22 THz circuit using nanocomputer numerical control (NCNC) presented very promising results. NCNC combined with UV lithography was also used by Shin *et al.* ^76^ in order to develop a Travelling wave tube circuit for high power and broadband terahertz applications. The circuit was fabricated with ~50 nm surface roughness and a cascaded nanocomposite cathode was synthesized, a fact which opens the way for the future development of watt-level terahertz radiation sources.

### Nanotechnology-supported new THz detectors

In the previous section, it was shown that QDs are very attractive for the generation of new THz sources. QDs could also innovate the existing sensors for detecting and sensing terahertz radiation. QDs have been shown to be able to detect single THz photons with or without the assistance of a magnetic field ^77^. Furthermore, carbon nanotube quantum dots can be used for developing highly sensitive detectors, which can also be frequency adjustable in the THz region ^78, 79^. Also, in a very recent publication, nanoscale carbon material was used for fabricating a tuneable quantum-dot sensing device ^80^. Additionally, QD-type detectors can expand the performance of THz detectors at temperatures where state-of-the art THz detectors have limited sensitivity ^81^. A novel sensor consisting of a QD (GaAS/AlGaAs QD), an electron reservoir and a superconducting single-electron transistor was presented with a cutting-edge performance ^82^. The detector operating at temperatures below 1 K was able to perform single-THz photon counting by relying on photon-to-plasmon and plasmon-to-charge conversion, followed by charge measurement in a single-shot mode.

As in the case of QDs, nanotubes/CNTs can also be used for detection purposes with several ways as nanoantenna ^71, 83^, bolometers ^84^ and even by using their mechanical properties ^85^. Furthermore, CNTs can be coated with bio-safe and biocompatible materials, like piezoelectric zinc oxide (ZnO), and be safely used as terahertz electromechanical nanoresonators ^72^. Their unique characteristics, like small junction areas, high electron mobilities and low estimated capacitances make them more attractive than solid-state components ^42^. CNTs, especially multi-walled CNTs, can be used as antennas in THz detectors since it has been shown that they interact with light in the same manner as simple dipole radio antennas ^83^. The polarisation and the length antenna effect are the key phenomena that can be used in optoelectronic devices. Furthermore, simulations have shown that CNT antenna arrays have better performance than single CNTs, and optimal design for receivers can be achieved. Very recently a novel resonant detector of THz radiation based on mechanically floating CNTs was presented ^85^. The detector consists of two electrically coupled single-wall-CNTs, which lie parallel over an insulator and are characterised by a proportional response of the plasma-mechanical resonance to the mechanical oscillation quality factor. The detector output signal depends on an AC displacement current that occurs between the CNTs due to plasma-mechanical oscillations of CNTs. Nanotubes can also be used as bolometers. Of course the need for new, better performing, detectors has not been driven only from the field of THz medical imaging. Astrophysicists studying the universe radiation at THz radiation require improvement of the sensitivity of current bolometers ^86^. To achieve this goal, the bolometers should be thermally isolated from the environment and have a very small capacity ^87^. The development of nanobolometers would deliver the required characteristics and enables high sensitivity of even single THz photons ^88^. The development of this kind of nanotechnology-based detectors pushes research to its current limits and possible future applications might be found in areas other than that of astronomy.

For some researchers, the development of novel electronic and semiconductor devices might improve and overcome many of the drawbacks that characterise current THz technology. In this direction, Balocco and his colleagues demonstrated novel planar nanodiodes, which are able both to emit and detect THz radiation at room temperature ^20^. The diode concept relies on asymmetric device nanochannels and showed high efficiency and speed sensitivity. Additionally, nanostructure physics principles and an antennae approach were used for fabricating a compact THz detector performing at room temperature ^89^. The inventors believed that these structures could contribute to the development of cost effective, compact and room-temperature operating THz emitters and detectors. One other significant limitation of current THz systems is that the detector and probe are not located close enough. As a consequence there is an affect on the sensitivity. New opportunities for high-resolution imaging can be achieved by integrating all the detection components on a semiconductor chip ^90^. In the future nanotechnology could facilitate this by providing the tools for minimising the dimensions of all the components of THz imaging set-ups. Generally, there are many perspectives on nanotechnology concerning terahertz electronics and many novel electronic components are expected to be developed ^91^.

### Nanotechnology for general THz purposes

In this section, it will be briefly discussed how nanotechnology can support THz imaging set-ups in areas that do not correspond directly to one of the previous discussed concepts (THz sources, detectors and contrast agents).

As already mentioned, terahertz radiation remained for many years unexplored and consequently a complete characterisation of its properties, physical characteristics and occurring phenomena have not yet been fully understood. An area that was affected by the absence of high-power THz sources is the study of the THz non-linear phenomena. In this direction, new sources that use nanotechnology will provide a useful tool for researchers in this field. Furthermore, other nanotechnology-based techniques could help the study of THz non-linear principles. For instance, nanostructures (nanoslits and nanogap split-ring resonators) were used in order to enhance the electric field and extend THz experiments into the non-linear regime ^92^. The understanding of the THz-related non-linear phenomena might allow their application for medical imaging or other biomedical purposes as it happens with the non-linear optical phenomena.

One important limitation of THz imaging techniques is the limited imaging resolution ^47^. One way that nanotechnology could help in minimising this drawback is the guide and focus of THz radiation by the use of SPPs on corrugated wires ^93^. In addition, THz propagation on wires is the key area for the development of probes for biological investigations. It has been demonstrated that propagation on wires with a size around a nanometre can be achieved ^94^. The small size of the wire opens the way for the development of MicroElectroMechanical Systems with sub-micrometre spatial resolution ^94^. These systems can be applied for the THz spectroscopy of biomolecules in biological entities. Other nanotechnology techniques can also be applied in the same direction for the collection of THz spectroscopic signatures from individual biological molecules. For instance, a single-electron source and a THz radiation detection cell can compose a coupled three-quantum-dot structure that can be applied for single-molecule spectroscopy studies ^95^. These novel THz spectroscopy techniques can be simultaneously used in the future with THz imaging methods.

### THz imaging for nanotechnology

The relationship between nanotechnology and THz is bidirectional, in the sense that the concurrent developments can contribute to both technologies. THz modalities have helped the expansion of nanotechnology. For instance, THz has made significant contributions in the study of semiconductor nanocrystals and quantum dots in recent years ^23^. It is widely believed that terahertz nanoscopy will advance the development of new novel nanostructures since it overcomes the limited spatial resolution due to the diffraction limits of the traditional optical microscopy techniques ^96^. Terahertz nanoscopy could be achieved by the development of novel probes for THz-scattering near-field optical microscopy (THZ-SNOM). The same concept has also been applied in the IR region with very promising results ^97^. In the scattering-type of SNOM, optical amplitude and phase images with nanoscale resolution are formed. In this technique, Atomic Force Microscope tips are illuminated with laser and the elastically scattering light is recorded interferometrically ^98^. Moreover, a resolution better than 40 nm was recently achieved by using IR and THz illumination ^96^, since the resolution depends on the sharpness of the tip and not to the wavelength. Compared with other imaging modalities, it has the advantage of rich spectral contrast and consequently it can provide information concerning chemical composition, structural status and conductivity ^99^. The possible applications of the technique are many and very promising. Recently it has been demonstrated that the method can be used for quantitative mapping of the local carrier concentration and mobility at the nanometre scale of different materials ^100^. Finally, one very interesting field concerning nanotechnology is the development of techniques and methods that can be used for detecting and tracking nanoparticles especially in human bodies. A recent publication showed that nanoscale metal barriers embedded in nanoslot antennas can be used to detect a single nanoparticle ^101^.

## Safety issues and ethical considerations

Although THz imaging set-ups use non-ionising radiation, risk issues must be considered since hazards can arise from a variety of mechanisms other than ionisation ^102^. Taking into account that emerging modalities are so new, it is obvious that a number of phenomena remain unexplored and their possible effects are unknown. These concepts are becoming even more complicated when nanotechnology-based techniques are used, since this innovative field is still in its infancy. The possible biological effects when electromagnetic (EM) radiation reacts with tissue include: thermal, acoustic, optical and photochemical mechanisms and their combinations ^25^. The importance of the effects that EM radiation might have in humans is highlighted by the number of international and national bodies that are interested in the guidelines in relation to its effects ^102^. A full and detailed understanding of the optical properties of tissues at THz radiation is necessary in order to achieve a safe *in vivo* image with THz imaging. Early studies have shown that the adsorption relies on the hydration of each tissue and tissues that are characterised by low water content possess a lower attenuation coefficient ^102, 103^. These results suggest that clinical imaging could be feasible only for certain applications and appropriate clinical protocols must be developed.

Initial safety analysis, based on the available guidelines for skin exposure to radiation of 15–115 GHz, determined the maximum permissible exposure (MPE) ^25, 103^. The results showed that, according to the currently available guidelines, THz imaging set-ups are safe. But it must be noted that the majority of the published guidelines concerning THz radiation take into account only the heating effects and ignore other possibly damaging effects, e.g. thermo-mechanical damage. Furthermore, the guidelines were established for specific exposure durations and these are not always appropriate for THz imaging purposes ^102^.

Research in the area is still in progress and as new applications are emerging, a full understanding of the THz-tissue interaction mechanism is imperative. For example, communication systems have started using frequencies of 300 GHz and above, which are not yet regulated ^104^. In order for THz radiation to be used as a biomedical tool, researchers are trying to develop compact THz spectrometers that can be used for measuring optical properties of biological tissues ^105^ and further investigations on the biological effects of THz radiation have been performed. Recently it has been shown that the 2.52 THz radiation generated primarily thermal effects in cells (fibroblasts) and thermal damage models can be used to predict the THz bioeffects ^106^. A detailed review of the current state of the research on the biological effects of THz radiation can be found in a recent review paper, where projects, official regulations and publications are summarised ^107^.

On the other hand, all medical nanotechnology-based techniques require special attention since possible risk, toxicity, ethical and social considerations must be taken into account ^2^. This area cannot be ignored since nanotechnology is a new and still unknown field. Shape, size and morphology play a significant role in bio-toxicity, since at low dimensions, the surface increases and as a result has higher reactivity. The hazards due to nanoparticle toxicity are crucial ^108^ and our current knowledge of the toxicity of the chemicals and materials is not sufficient when materials have dimensions in the nanoscale ^109^. Moreover, due to their small size nanoparticles can penetrate humans through three paths: skin, breathing (mouth and/or nose) and digestive system (mouth) ^110^. None of these three possible ways has been well studied at the moment. The risk of nanoparticles does not appear only during their application but also in every stage of their cycle from their production to transfer ^111, 112^, while possible environment pollution cannot be ignored ^113– 115^. There is evidence that nanoparticles are responsible for unusual diseases ^116^ and what is more, the physical and biological mechanisms that are involved when nanoparticles are exposed to any kind of radiation within biological tissues remain unknown. Nonetheless, there is still a huge absence of clear regulatory guidelines, safety standards and MPE approvals for almost all the nanotechnology-related techniques ^117^. Concerning regulation, the history of previous technologies must be taken into account so as to avoid repeating past mistakes ^118^. Since nanotechnology is still in its infancy, new risks, ethical challenges and issues related with privacy and justice will arise while nanotechnology moves from research to clinical practice ^117^. Finally, a crucial point has to do with how society will react and how willing it is to accept an innovative technology that has not yet proved to be either effective or safe ^119^.

## Conclusions

Throughout this review, we have shown that nanotechnology can support emerging THz imaging modality in order to overcome some of its limitations. This can be achieved in many nanotechnology-based techniques and several drawbacks can be overcome, from the low performance of the emitting source to the miniaturisation of the whole set-up. These research results indicate that nanotechnology could help in the development of high-resolution, sensitive and portable detectors and new efficient sources for THz imaging purposes. What is more, the use of nanoparticles as contrast agents can enhance THz signals and detection, not only from healthy areas but also from specific pathological areas such as tumours. Although the techniques are in their infancy, it seems possible that nanotechnology may be applied to help THz imaging modality find its way into real everyday clinical use.
