# Supplementary material for: Nanotechnology-supported THz medical imaging
Source: F1000Res. 2013 Mar 28;2:100. [Version 1] doi: 10.12688/f1000research.2-100.v1 (PMC3869482; doi:10.12688/f1000research.2-100.v1)
Supplement: Peer review history

# Peer review: Referee response for version 1

**Published:** 2013-07-16

**Authors:** Safieddin Safavi-Naeini

**Affiliations:** 1 Electrical and Computer Engineering Department, University of Waterloo, Waterloo, ON, USA

The manuscript is a general review of nano-technology as applied to THz system for medical imaging. The review could have gone deeper than just a brief summary of other works, which is useful but not sufficient. The manuscript would be much more useful and informative if the authors would include a brief and simple explanation of some important THz imaging modalities by including a general block diagram of the system. 

The analytical content of the manuscript could be improved substantially by including a more rigorous description of various techniques and their assessment. The entire article only has 2 or 3 very general pictures which do not describe any specific technology or approach at any depth. More pictures, plots, and tables should be added to support authors’ arguments.

The conclusions are very general and only based on summary of the other articles. No analytical method and independent data and/or results generated by the authors have been provided.

I have read this submission. I believe that I have an appropriate level of expertise to confirm that it is of an acceptable scientific standard.

# Peer review: Referee response for version 1

**Published:** 2013-06-04

**Authors:** Creidhe O’Sullivan

**Affiliations:** 1 Department of Experimental Physics, National University of Ireland, Maynooth, Ireland

This article provides not only a useful review of the ways in which nanotechnology can contribute to the field of THz medical imaging but also a review of the possibilities and shortcomings of the field in general. I believe that it will be of interest to both nanotechnology and THz researchers in general.  A good list of references is provided. The authors concentrate on issues related to contrast agents, sources and detectors and mention many of the most widely-used and promising of these. Highlighting the potential impact of nanotechnology marks this review out from other THz review articles. 

The abstract and conclusion sections are appropriate and provide a useful summary for anyone briefly scanning the article. The publication search method used by the authors is clearly described.

Minor comments

- The authors switch between THz and terahertz throughout the article.
- On page 4 (3rd paragraph). I find the sentence *“The THz imaging systems can be separated into two main categories: Passive (also named incoherent) and Active (also named coherent) Pulsed or Continuous”* confusing. Which are the 2 categories? Are all passive systems incoherent and pulsed? etc. Incoherent and coherent refer to the detection of the radiation, not whether the imaging set-up is active or passive. Should it be phrased *“THz imaging systems can be separated according to whether they are active or passive, incoherent or coherent, continuous or pulsed”.*
- Page 4 2nd column, 2nd paragraph *“.. separated depending on whether …”* or *“separated on the basis of...”  *Would it be worth a line in the text (it is in table 2) that an advantage to THz is that it can propagate through materials such as bandages.
- Towards end of p.8 change " *capacity" * to " *heat capacity*".
- In the Conclusion, 1st line, *“..emerging THz imaging modality..." *should be changed to “ *..emerging THz imaging modalities.”* 

I have read this submission. I believe that I have an appropriate level of expertise to confirm that it is of an acceptable scientific standard.
